# Supplementary material for: Insights into degradation mechanism of N-end rule substrates by p62/SQSTM1 autophagy adapter
Source: Nat Commun. 2018 Aug 17;9:3291. doi: 10.1038/s41467-018-05825-x (PMC6098011; doi:10.1038/s41467-018-05825-x)
Supplement: Supplementary file 1 — Supplementary Information [file 41467_2018_5825_MOESM1_ESM.pdf]

Supplementary Figures and Tables

Insights into degradation mechanism of N-end rule substrates by p62/SQSTM1 autophagy adaptor

D. H. Kwon *et al.*

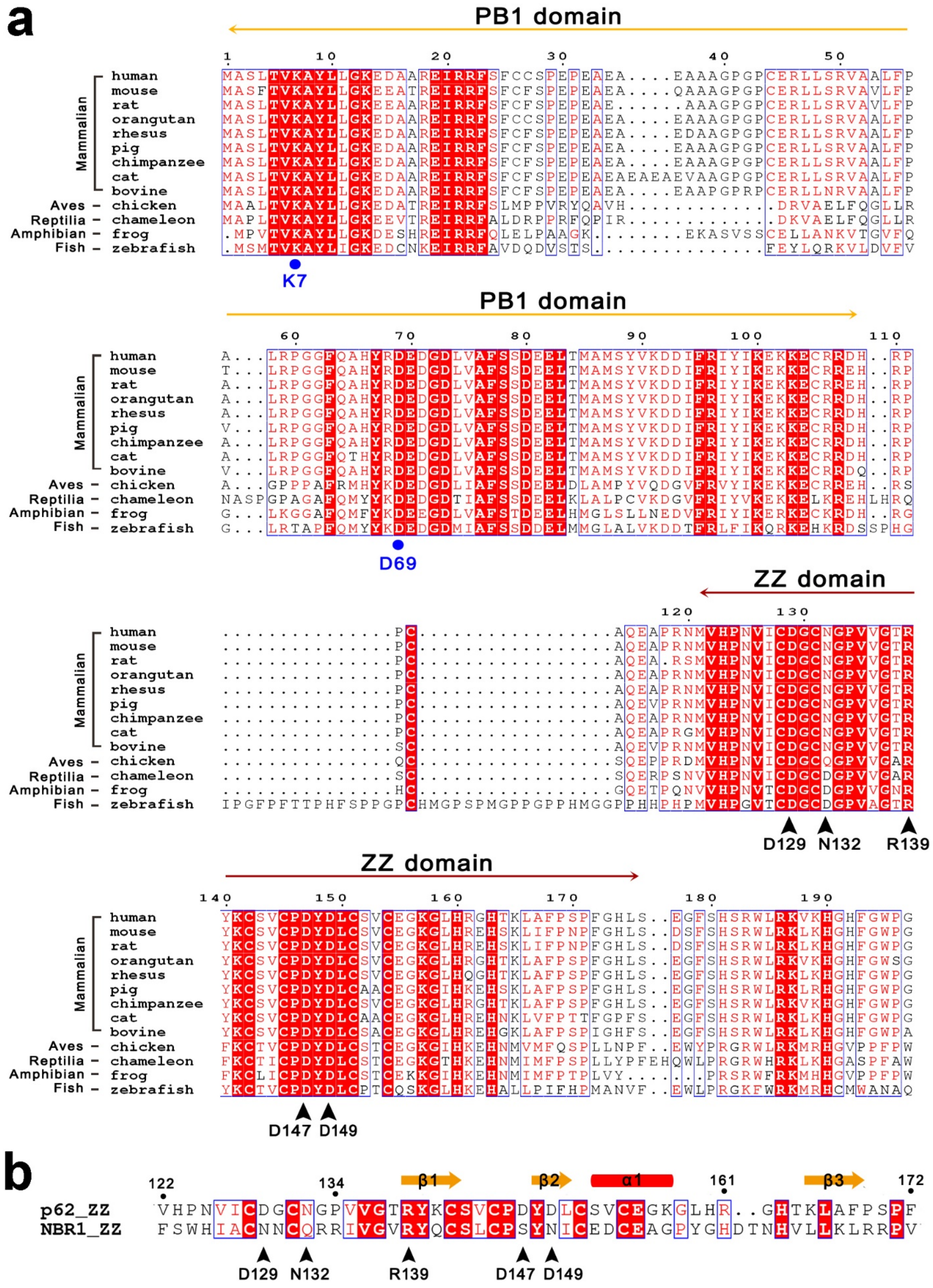
a
PB1 domain
Mammalian
human
mouse
rat
orangutan
rhesus
pig
chimpanzee
cat
bovine
Aves – chicken
Reptilia – chameleon
Amphibian – frog
Fish – zebrafish
K7
D69
ZZ domain
D129
N132
R139
D147
D149
b
p62_ZZ
NBR1_ZZ
β1
β2
α1
β3
122
134
161
172

Supplementary Figure 1. Sequence alignment of p62

(**a**) Sequence alignment of PB1 and the ZZ-domain of p62 orthologs. Organism sources are indicated on the left. The domain boundary of PB1 and ZZ is indicated above the sequence alignment. Every 10th residue of p62 from human (first row) is marked with a dot and the corresponding residue number. Mutated residues K7 and D69 utilized for disrupting oligomerization of p62 are marked by blue filled circle. (**b**) Sequence comparison of the ZZ-domain between p62 and NBR1 from human. Secondary structural elements (α-helix as a red cylinder and β-strands as orange arrows) are shown above the sequence alignment. Key determinants for recognizing N-degrons are marked by black arrows. Red shading and blue boxes indicate residues that are identical and highly conserved in all sequences.

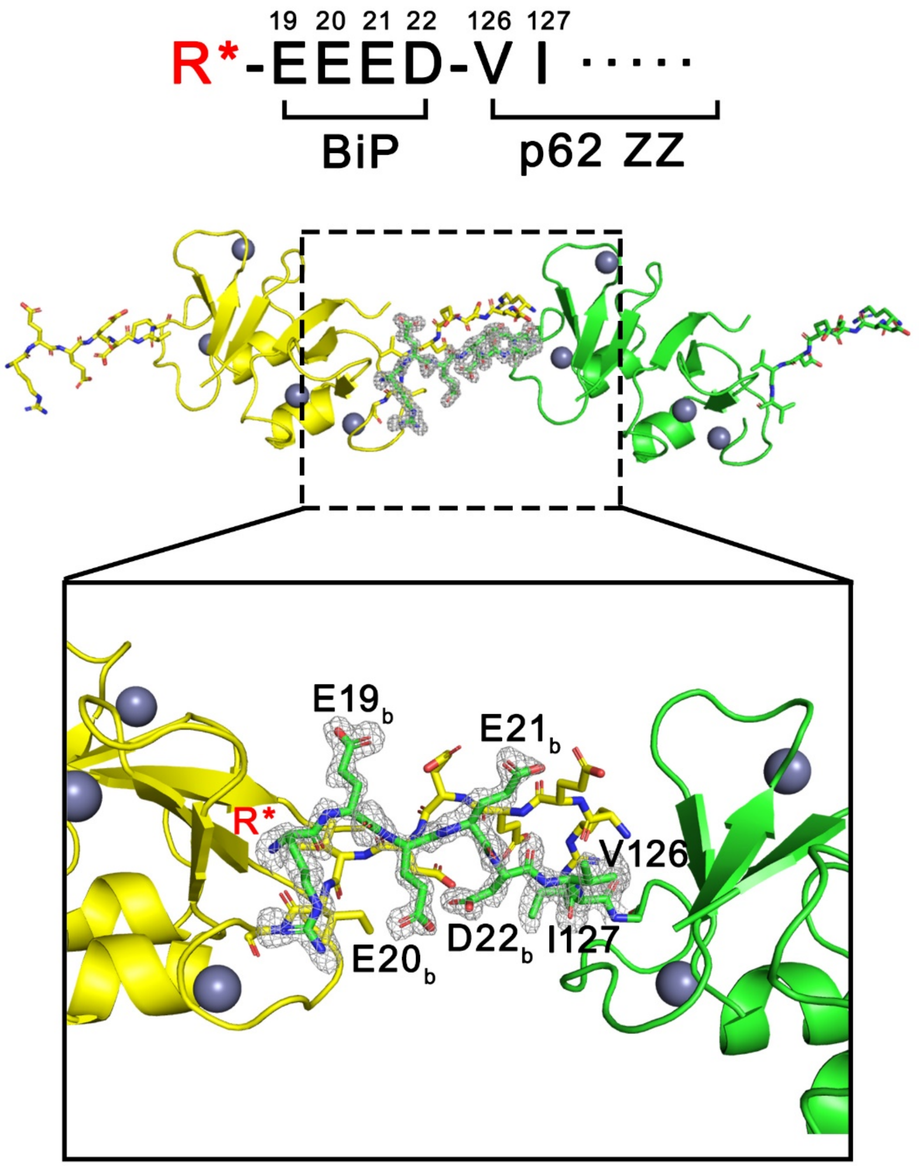

Supplementary Figure 2. Binding of the N-degron sequence of an adjacent R-BiP-ZZ fusion protein to the recognition site of the ZZ-domain.

For the N-degron bound structure, five R-BiP residues were covalently attached to the N-terminus of the ZZ-domain (residues Val126 to Phe172). Crystal packing showing the N-terminal residues of the green symmetry equivalent molecule bound to the active site of the yellow molecule. The dotted boxed region is enlarged and the 2Fo-Fc electron density map contoured at 1.2 σ is shown with a stick model of the N-degron sequence.

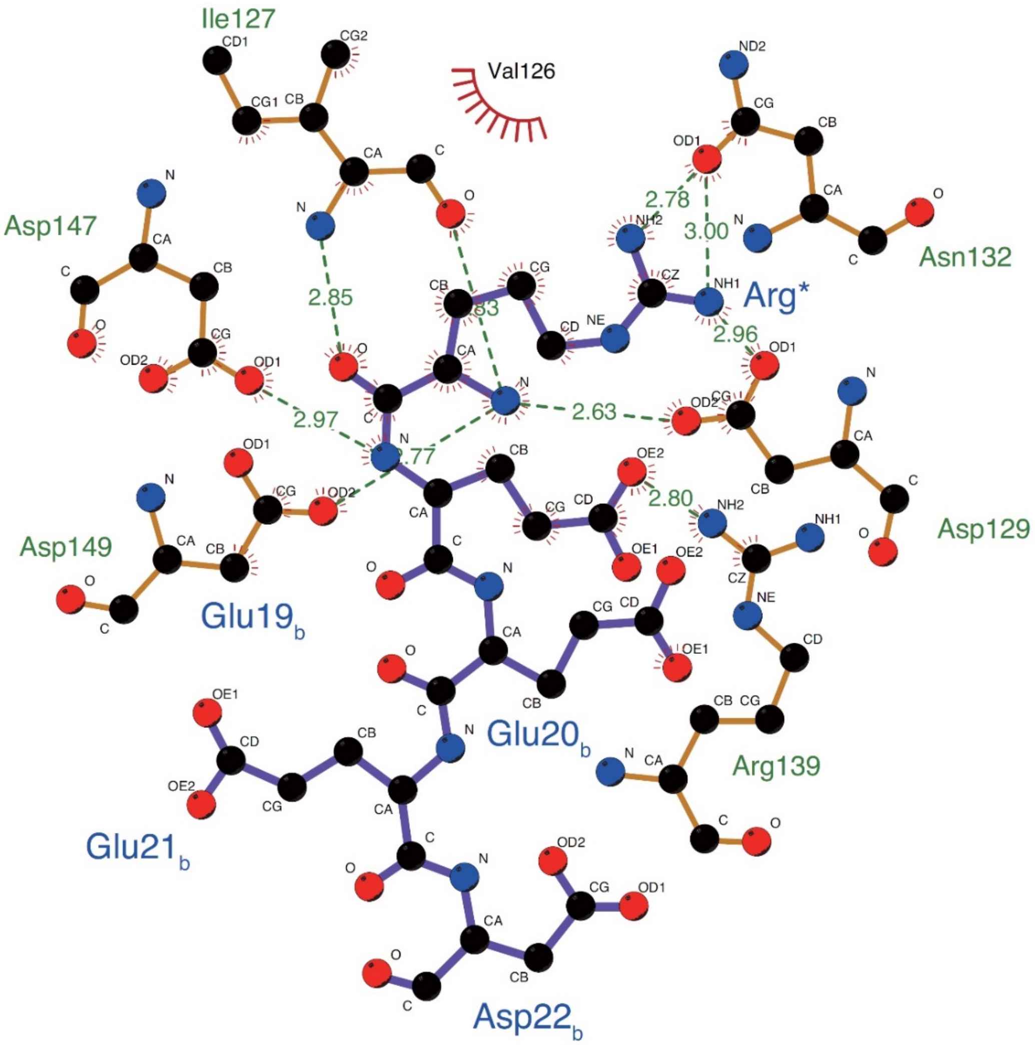

Supplementary Figure 3. Schematic of interactions between the ZZ-domain and R-BiP N-degron

Hydrophobic interactions are indicated with red starbursts, and hydrogen bonding interactions are shown as green dashed lines with hydrogen bonding distances. The ZZ-domain and N-degron residues are labeled green and blue, respectively. For R-BiP sequence, * and subscript ‘b’ are attached for clarity.

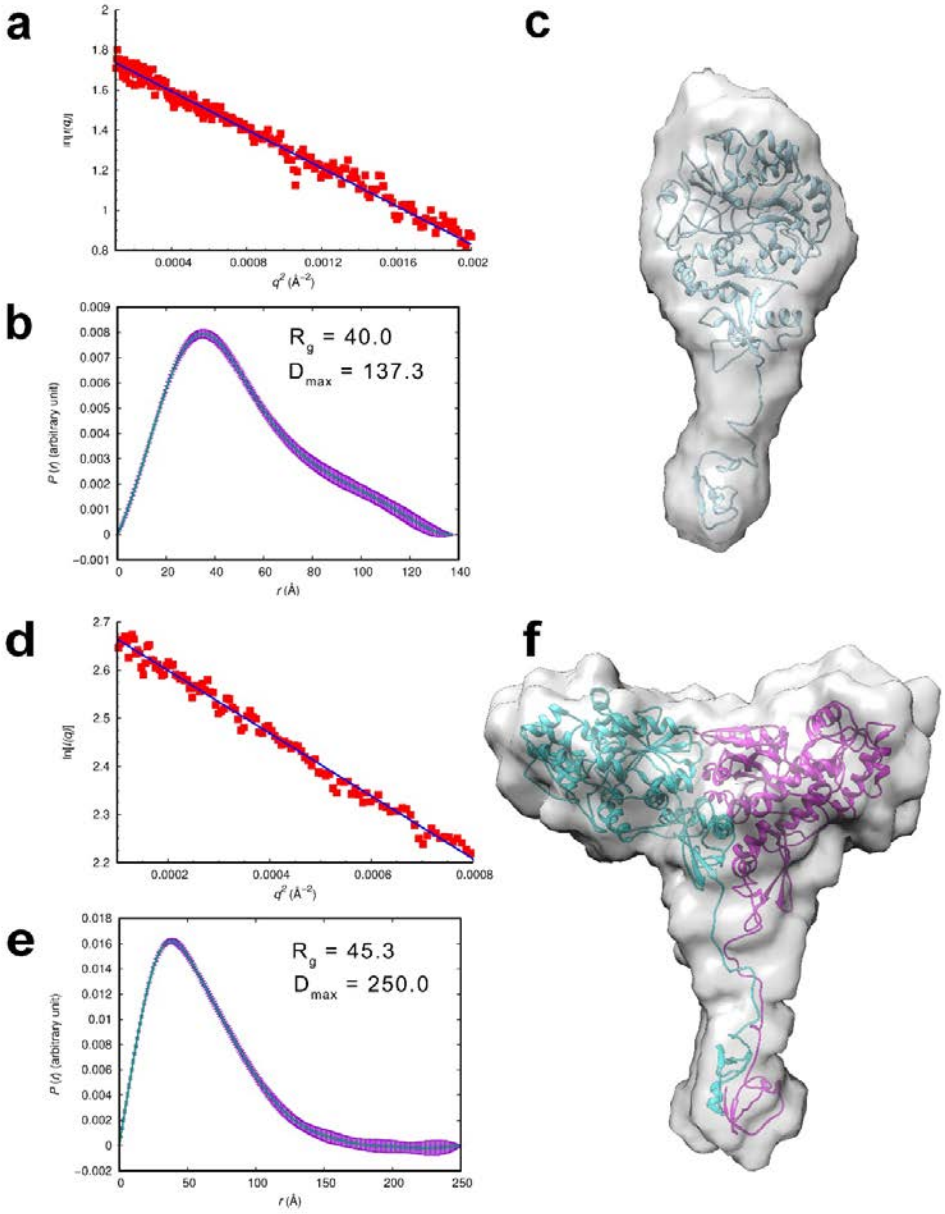

Supplementary Figure 4. Solution structures of MBP-PB1-ZZ D69A mutant

SAXS data analysis of monomeric (**a-c**) and dimeric (**d-f**) forms of MBP-PB1-ZZ mutant D69A. (**a,d**) SAXS experimental scattering curve (red) with theoretical curve (blue) fit by DAMMIF. (**b,e**) Pair distance distribution function, $P(r)$ with error bar (violet) showing D_{max} and R_g values. (**c,f**) Molecular envelope (gray) from DAMMIF with a fitted model (monomer: cyan, dimer: cyan and magenta) using the Molecular Dynamics Flexible Fitting method. The initial structure of MBP-PB1-ZZ was established by combining MBP (PDB ID: 5JST [https://www.rcsb.org/structure/5JST]), p62 PB1 (PDB ID: 4MJS [https://www.rcsb.org/structure/4MJS]), linker (modeled by Chimera) and the ZZ-domain (PDB ID: 5YP7) using Chimera (UCSF).

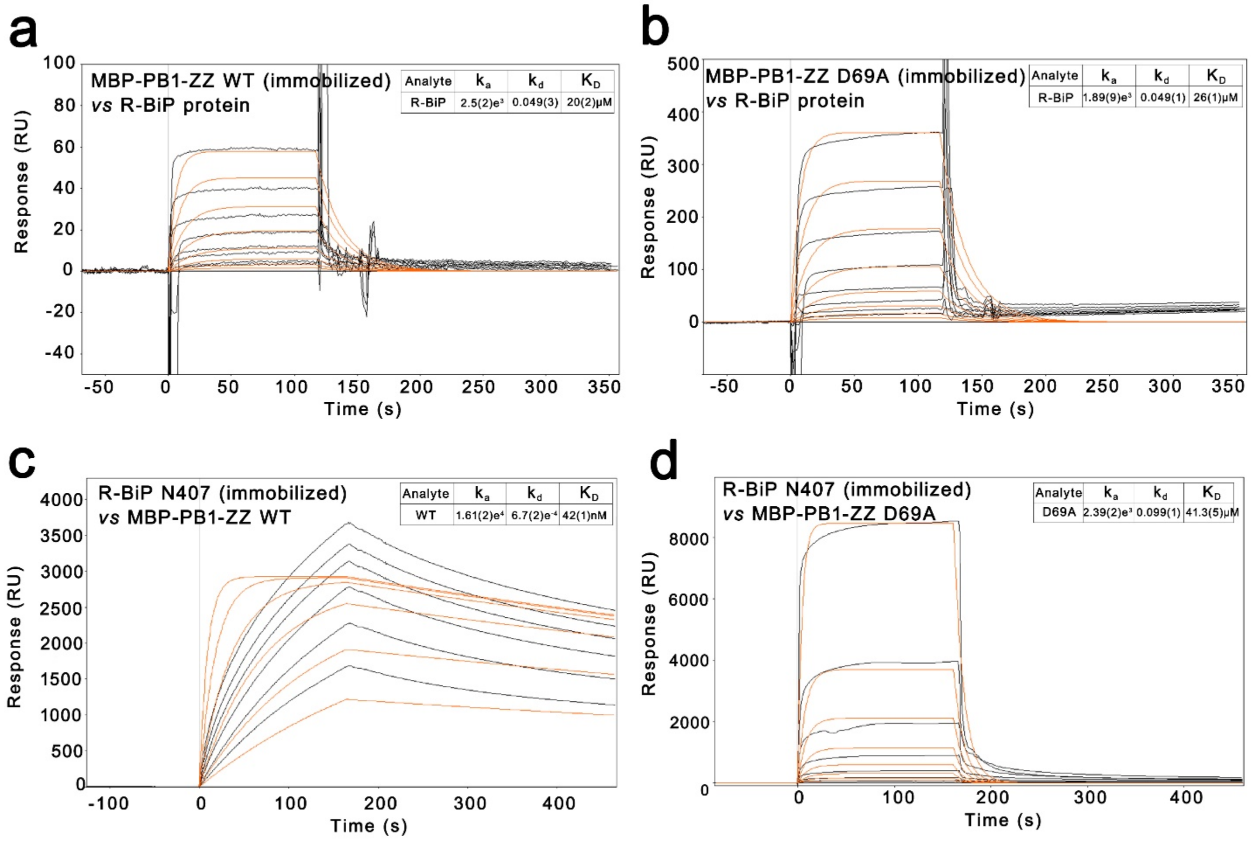

Supplementary Figure 5. Binding affinity measurements between p62 and R-BiP protein using SPR

Surface plasmon resonance (SPR) sensorgram of R-BiP N407 protein (R*-19~407 construct) binding to immobilized MBP-PB1-ZZ WT (**a**) and MBP-PB1-ZZ P69A mutant (**b**) on the CM5 chip. Conversely, the SPR sensorgram of the binding of p62 WT (**c**) and D69A mutant (**d**) to immobilized R-BiP protein on the CM5 chip. Response (RU, resonance unit) is plotted against time. The K_D was derived from at least 5 different concentrations of ligand.

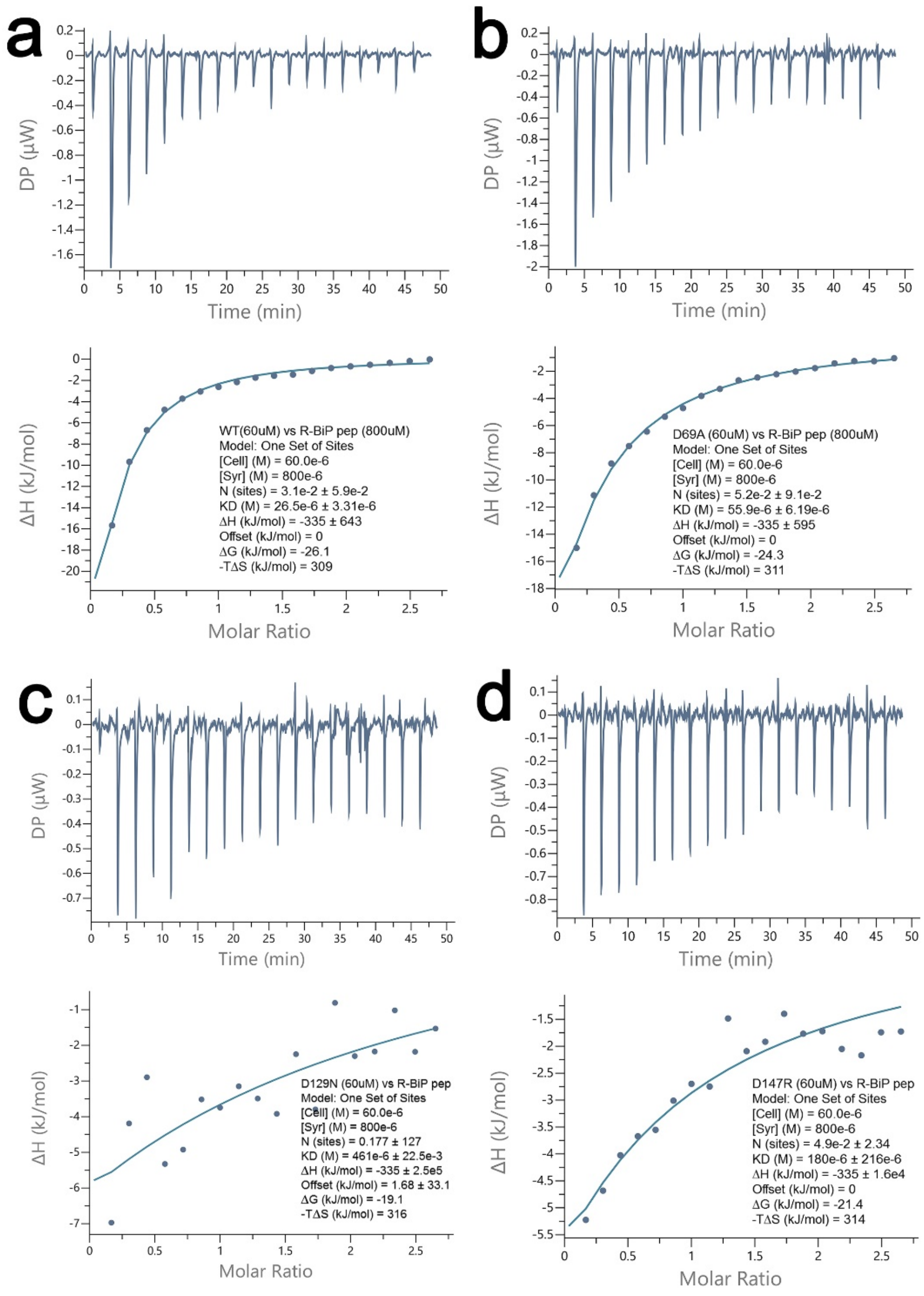

Supplementary Figure 6. Binding affinity measurements between p62 and R-BiP peptide using ITC

Measurement of the affinities of p62 WT (**a**) and D69A mutant (**b**) to R-BiP peptide using ITC. The K_D values of the WT and D69A mutant with N-degron peptide were 26.5 ± 3.31 μM and 55.9 ± 6.19 μM, respectively, and showed unusual binding stoichiometry. ITC measurement of the binding of D129N (**c**) and D147R (**d**) mutants to N-degron peptide. The K_D values were much higher than those of WT and monomeric mutant, which confirms the binding defect of these mutants.

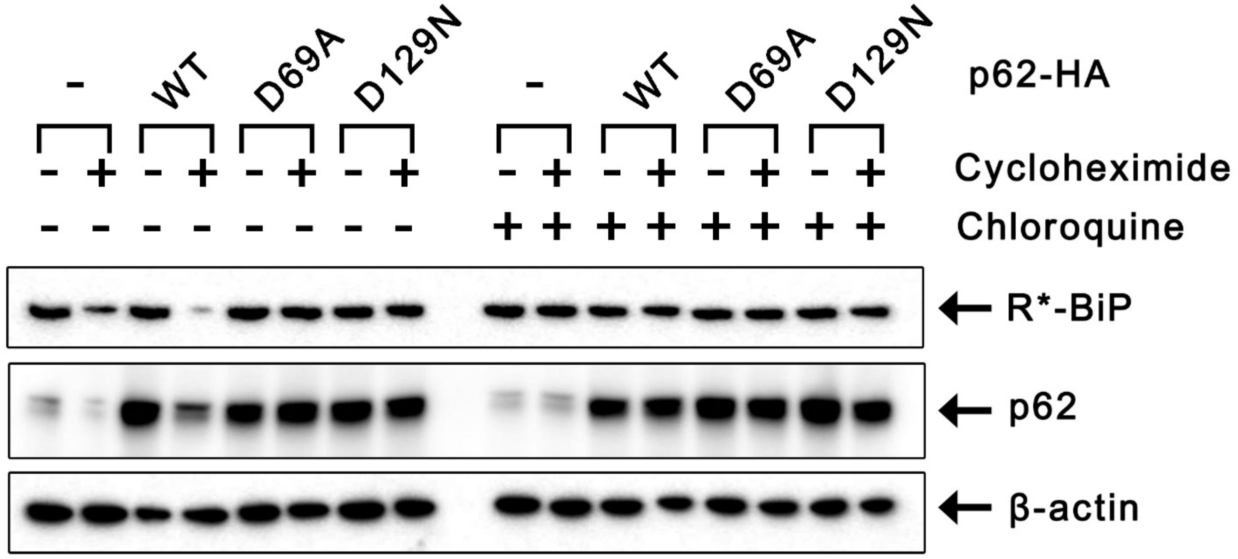

Supplementary Figure 7. Degradation assay of R-BiP and p62 protein

Degradation assay of R-BiP generated from Ub–R-BiP using oligomerization defect D69A and N-degron recognition defect D129N mutant in HeLa cells in the absence of MG132, which showed the same results as shown in Figs. 2e and 3b. To visualize p62 co-degradation, the same experiments were performed with reduced amounts of p62-HA DNA (0.5 μg) and prolonged treatment with 50 μg/ml cycloheximide (16 hr). p62 WT underwent significant co-degradation with R-BiP protein, unlike the case with the D69A and D129N mutants. The degradation of R-BiP protein and p62 was blocked in presence of autophagy inhibitor, chloroquine (30 μM, 16 hr).

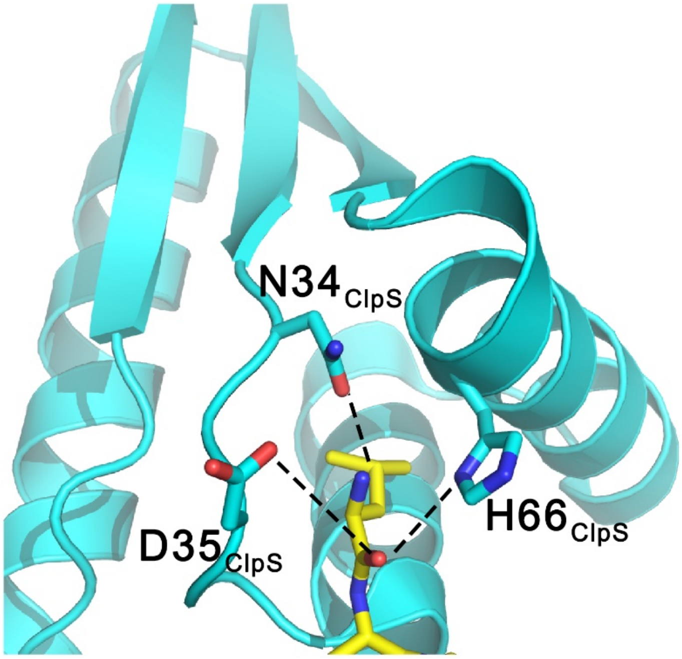

Supplementary Figure 8. Recognition of type-2 N-degron by ClpS

Ribbon diagram of ClpS from *E. coli* in complex with Leu-peptide substrate. The N-degron and key determinant residues in ClpS are shown as stick models. The hydrogen bonding interactions are indicated as black dashed lines. The α-amino group of the N-degron is recognized by the side chain of Asn34 from ClpS.

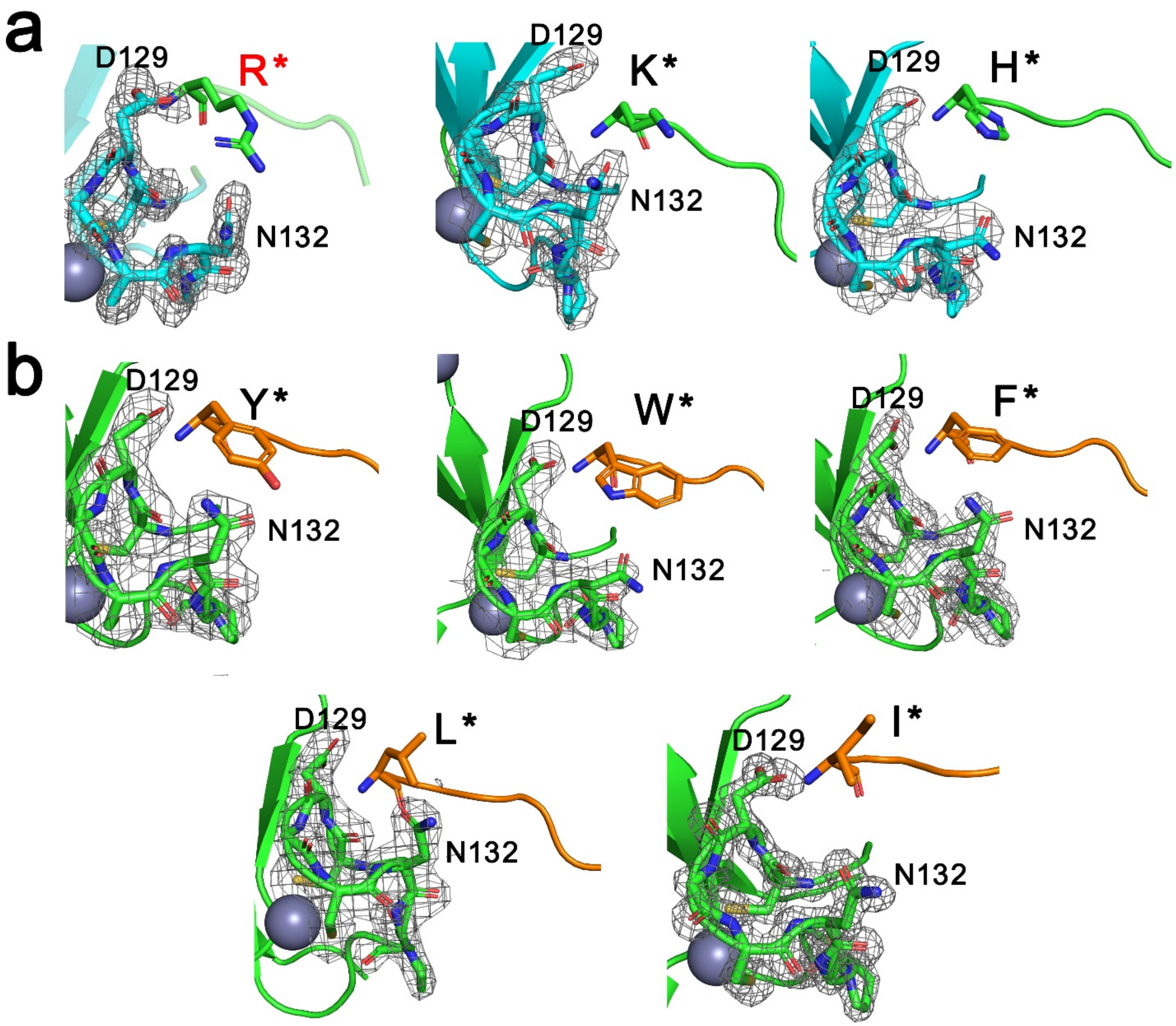

Supplementary Figure 9. Electron density map around the N-degron recognition region in the ZZ-domain of p62

(**a**) Electron density map of the ZZ-domain of p62 showing the recognition of type-1 N-degrons. The ZZ-domain and type-1 N-degrons are colored cyan and green, respectively. (**b**) Electron density map of the ZZ-domain of p62 showing the recognition of type-2 N-degrons. The ZZ-domain and N-degrons are colored green and dark orange, respectively. 2Fo-Fc electron density map contoured at 1.2 σ using the data ranging from 1.5 to 2.9 Å resolution. The bound peptide and key residues of p62 are shown as stick models and are labeled. Nitrogen and oxygen atoms are colored blue and red, respectively. The important Asn132 residue of p62 involved in the recognition of both type-1 and type-2 N-degron substrates shows clear electron density.

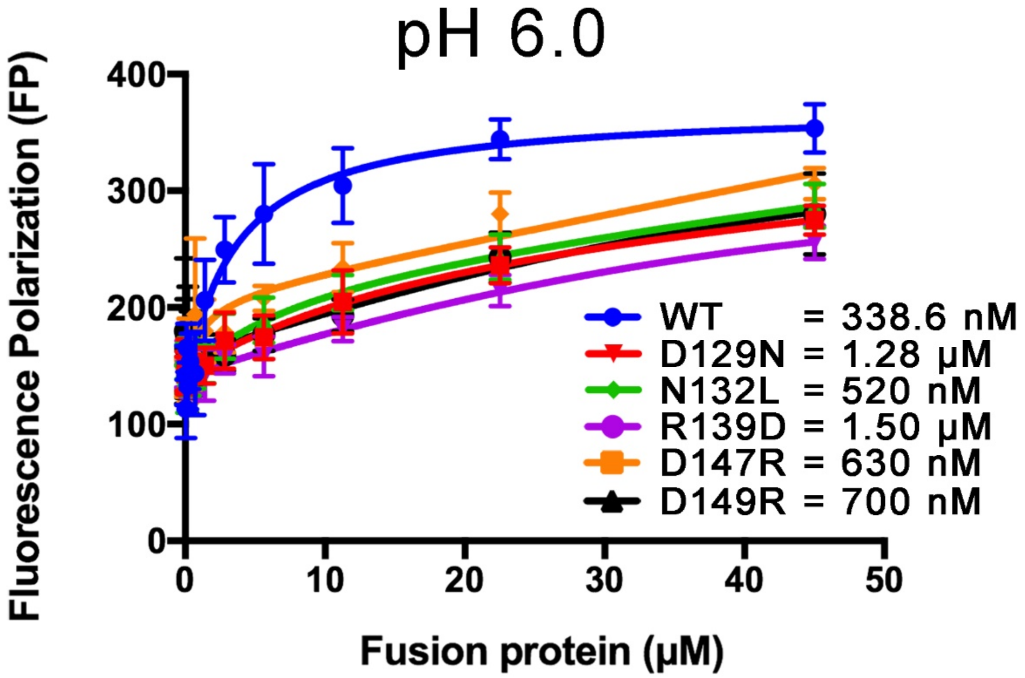

Supplementary Figure 10. Mutational effects of key determinants on the recognition of N-degrons at low pH

Binding affinity measurements using FITC-labeled R-BiP peptide against increasing concentrations of p62 mutants (MBP-PB1-ZZ WT – blue line, D129N – red line, N132L – green line, R139D – violet line, D147R – orange line and D149R – black line) at pH 6.0. The error bars represent standard error of the mean (S.E.M.) of more than three independent experiments.

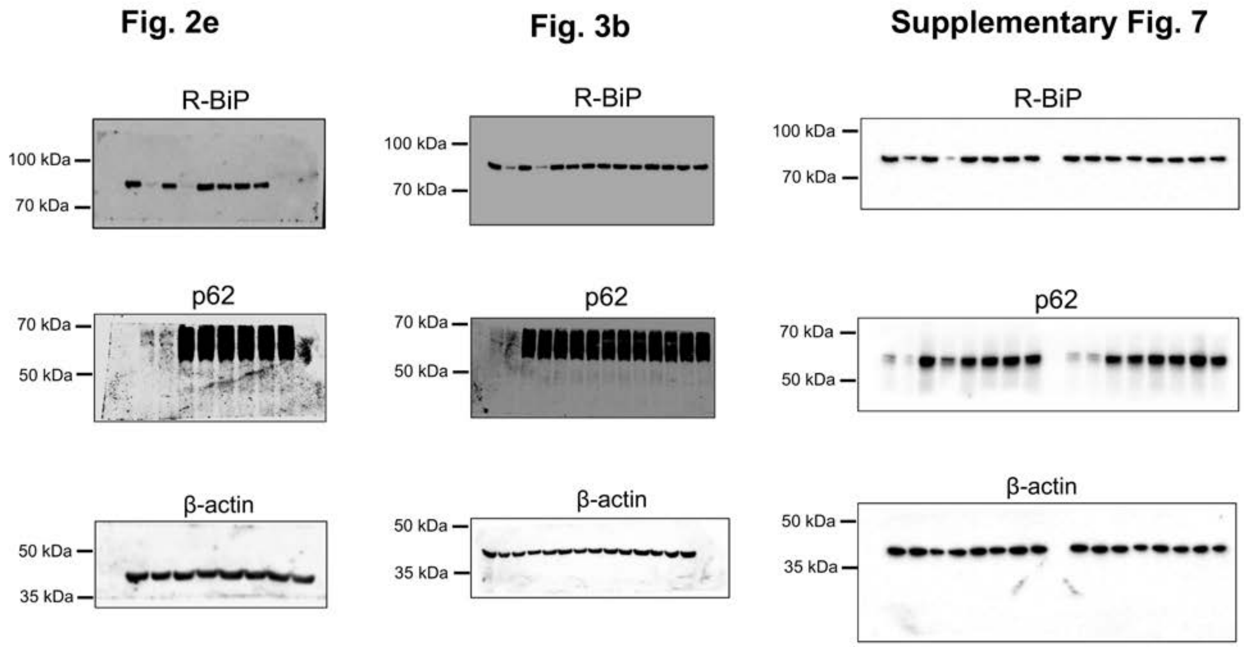

Supplementary Figure 11. Uncropped images of Western blots
Related to Fig. 2e, Fig. 3b and Supplementary Fig. 7 as indicated.

Supplementary Table 1. Data collection, phasing and refinement statistics of the ZZ-domain of p62

**Data collection**	Apo	REEED_Zn (Peak)
Space group	$P\ 2_1\ 2_1\ 2$	$P\ 2_1\ 2_1\ 2_1$
Cell parameters		
a, b, c (Å)	54.0, 43.3, 34.7	29.0, 44.0, 66.6
α, β, γ (°)	90, 90, 90	90, 90, 90
Wavelength (Å)	0.90000	1.282282
Resolution (Å)[a]	33.8 - 1.42 (1.47 - 1.42)	50.0 - 1.77 (1.80 - 1.77)
R_{merge}	0.069 (0.824)	0.066 (0.803)
$I / \sigma (I)$	41.9 (2.55)	45.45 (2.97)
Completeness (%)	99.0 (99.2)	98.9 (95.7)
Redundancy	5.9 (5.2)	6.2 (5.9)
**SAD-Phasing**		
No. of Zn atoms		4
Initial figure of merit (FOM)		0.35
FOM after density modification		0.61
**Refinement**		
Resolution (Å)	33.82 - 1.42 (1.47 - 1.42)	
No. reflections	15,680 (1,509)	
R_{work} / R_{free} (%)	18.8 / 20.9	
No. atoms	737	
Protein	648	
Zn^{2+} / Water	4 / 85	
B-factors ($Å^2$)	29.88	
Protein	28.40	
Zn^{2+} / Water	21.16 / 41.60	
R.M.S deviations		
Bond length (Å)	0.010	
Bond angles (°)	1.32	
Ramachandran statistics		
Favored	100	
Allowed	0	
Outliers	0	
**PDB ID**	5YP7	

[a] Values in parentheses are for highest-resolution shell.

Supplementary Table 2. Data collection and refinement statistics of the ZZ-domain of p62 in complex with type-1 N-degrons

**Data collection**	REEED	KEEED	HEEED
Space group	*P* 2_1 2_1 2_1	*P* 2_1 2_1 2_1	*I 2 3*
Cell dimensions			
a, b, c (Å)	29.0, 44.0, 66.6	29.1, 43.8, 67.2	114.0, 114.0, 114.0
α, β, γ (°)	90, 90, 90	90, 90, 90	90, 90, 90
Wavelength (Å)	0.90000	1.2000	1.0000
Resolution (Å)[a]	19.8 - 1.45	21.9 - 2.50	33.7 - 2.90
	(1.5 - 1.45)	(2.59 - 2.50)	(2.95 - 2.90)
R_{merge}	0.061 (0.759)	0.168 (0.533)	0.110 (0.663)
$I / \sigma (I)$	49.5 (4.02)	13 (2.05)	31.4 (5.14)
Completeness (%)	97.3 (98.7)	96.1 (88.6)	99.89 (100.00)
Redundancy	6.6 (6.6)	4.5 (3.2)	13.1 (13.4)
**Refinement**			
Resolution (Å)	19.8 - 1.45	21.91 - 2.50	36.04 - 2.5
	(1.5 - 1.45)	(2.59 - 2.50)	(2.59 - 2.5)
No. reflections	15,274 (1,500)	2,939 (236)	8,701 (882)
R_{work} / R_{free} (%)	16.1 / 19.6	23.9 / 26.7	25.2 / 29.4
No. atoms	858	792	1,416
Protein	777	762	1,386
Zn^{2+} / Water	4 / 77	4 / 26	8 / 22
B-factors (Å^2)	34.28	35.68	54.56
Protein	33.19	35.80	54.68
Zn^{2+} / Water	22.12 / 45.53	31.56 / 32.71	56.26 / 46.31
R.M.S deviations			
Bond length (Å)	0.011	0.007	0.006
Bond angles (°)	1.09	0.69	0.59
Ramachandran statistics			
Favored	98.99	97.96	95.51
Allowed	1.01	1.02	4.49
Outliers	0	1.02	0
**PDB ID**	5YP8	5YPA	5YPB

[a] Values in parentheses are for highest-resolution shell.

Supplementary Table 3. SAXS data collection and analysis

**Data collection parameters**		
Instrument	PAL 4C1	
Beam geometry	20 mm slit	
Wavelength (Å)	1.24	
q range ($Å^{-1}$)	0.001 ~ 0.2	
Exposure time (second)	5	
Concentration range (mg ml^{-1})	1.0 ~ 11.0	
Temperature (K)	289	
**Structural parameters**	Monomer[a]	Dimer[b]
$I(0)$ (cm^{-1}) [From $P(r)$]	6.03	15.43
R_g (Å) [From $P(r)$]	40.07	45.30
$I(0)$ (cm^{-1}) [From Guinier]	6.03	15.43
R_g (Å) [From Guinier]	40.02	45.31
D_{max} (Å)	132.73	149.55
Porod volume estimate, V_p ($Å^3$)	66,700	156,000
Dry volume calculated from monomer sequence	76,731	76,731
Molecular-mass determination		
Molecular mass M_r [From Porod volume (V_p*0.66)]	44.0 kDa	103.0 kDa
Calculated monomeric M_r from monomer sequence	63.4 kDa	63.4 kDa
**Software employed**		
Primary data reduction	In-house program	
Data processing	PRIMUS	
Ab initio analysis	DAMMIF	
Validation and averaging	DAMAVER	
Rigid-body modeling	Situs Program Suite	
Computation of model intensities	Chimera	

[a] Monomeric species of MBP-PB1-ZZ D69A (2.5 mg/ml) at pH = 8.0.

[b] Dimeric species of MBP-PB1-ZZ D69A (5.0 mg/ml) at pH = 8.0.

Supplementary Table 4. Data collection and refinement statistics of the ZZ-domain of p62 in complex with type-2 N-degrons

**Data collection**	FEEED	YEEED	WEEED	LEEED	IEEED
Space group	*C 1 2 1*	*I 2 3*	*I 2 3*	$P\ 2_1\ 2_1\ 2_1$	$P\ 1\ 2_1\ 1$
Cell dimensions					
a, b, c (Å)	34.7, 43.3 ,54.0	114.5, 114.5, 114.5	115.0, 115.0, 115.0	28.5, 44.3, 68.2	33.7, 33.4, 35.0
α, β, γ (°)	94.81, 46.29, 54.88	90, 90, 90	90, 90, 90	90, 90, 90	90, 103.61, 90
Wavelength (Å)	0.90000	1.0000	0.90000	1.0000	1.20000
Resolution (Å)[a]	31.4 - 1.96	36.2 - 2.85	40.7 - 2.95	27.0 - 2.20	17.9 - 1.62
	(2.03 - 1.96)	(2.95 - 2.85)	(3.05 - 2.95)	(2.27 - 2.20)	(1.68 - 1.62)
R_{merge}	0.082 (0.559)	0.183 (0.804)	0.082 (0.816)	0.115 (0.534)	0.040 (0.165)
$I / \sigma (I)$	24.6 (1.68)	20.5 (3.00)	50.0 (4.45)	25.3 (4.67)	34.7 (7.78)
Completeness (%)	93.5 (79.0)	100 (100)	99.9 (100)	98.3 (99.1)	94 (86.8)
Redundancy	3.2 (2.8)	11.4 (10.9)	10.6 (10.9)	5.2 (5.4)	3.3 (2.9)
**Refinement**					
Resolution (Å)	31.4 - 1.96	36.2 - 2.85	40.7 - 2.95	27.0 - 2.20	17.9 - 1.63
	(2.03 - 1.96)	(2.95 - 2.85)	(3.05 - 2.95)	(2.27 - 2.20)	(1.68 - 1.62)
No. reflections	15,737 (1,527)	5,991 (596)	5,455 (543)	4,633 (429)	9,010 (835)
R_{work} / R_{free} (%)	20.2 / 23.0	23.6 / 27.4	22.6 / 26.1	25.5 / 28.5	14.5 / 18.1
No. atoms	1,584	1,420	1,429	774	868
Protein	1,513	1,412	1,421	760	747
Zn^{2+} / Water	8 / 63	8 / -	8 / -	4 / 10	4 / 117
B-factors (Å^2)	43.39	44.85	71.21	41.44	15.45
Protein	43.30	44.87	71.22	41.44	13.80
Zn^{2+} / Water	37.24 / 46.25	42.23 / -	69.2 / -	39.21 / 42.92	12.26 / 23.72
R.M.S deviations					
Bond length (Å)	0.005	0.003	0.004	0.002	0.017
Bond angles (°)	0.89	0.58	0.71	0.61	1.64
Ramachandran statistics					
Favored	96.34	92.78	93.37	96.94	96.88
Allowed	3.66	7.22	6.63	3.06	3.12
Outliers	0	0	0	0	0
**PDB ID**	5YPC	5YPE	5YPF	5YPG	5YPH

[a] Values in parentheses are for highest-resolution shell.

Supplementary Table 5. Primers used in this study

Primer	Sequence	Usage
hSqstm1 BglII F	cagg aga tct atg gcg tcg ctc acc gtg aag	Full-length gene cloning
hSqstm1 EcoRI R	cgtg gaa ttc tca caa cgg cgg ggg atg ctt	Full-length gene cloning
hSqstm1 Nd126 F	ctg gtt ccg aga tct atc tgc gat ggc tgc	N-terminus deletion (1-126)
hSqstm1 Nd126 R	gca gcc atc gca gat aga tct cgg aac cag	N-terminus deletion (1-126)
hSqstm1 K7A F	gcg tcg ctc acc gtg gcg gcc tac ctt ctg ggc	Lys 7 to Ala 7 mutation
hSqstm1 K7A R	gcc cag aag gta ggc cgc cac ggt gag cga cgc	Lys 7 to Ala 7 mutation
hSqstm1 D69A F	cag gcg cac tac cgc gct gag gac ggg gac ttg	Asp 69 to Ala 69 mutation
hSqstm1 D69A R	caa gtc ccc gtc ctc agc gcg gta gtg cgc ctg	Asp 69 to Ala 69 mutation
hSqstm1 BamHI 128 F	aat gga tcc tgc gat ggc tgc aat ggg cct gtg	crystallization construct gene
hSqstm1 EcoRI F	cttg gaa ttc atg gcg tcg ctc acc gtg aag	Full-length gene cloning
hSqstm1 XhoI R	gtg ctc gag tca caa cgg cgg ggg atg ctt	Full-length gene cloning
hSqstm1 EcoRI F	att gaa ttc atg gcg tcg ctc acc gtg aag	Full-length gene cloning
LC3B BamHI F	ccg gga tcc atg ccg tcg gag aag acc ttc aag	LC3B fuson construct generating
hSqstm1 172 XhoI R	ata ctc gag cta gaa ggg gct ggg gaa tgc ga	crystallization construct gene
hSqstm1 FEEED F	AG GAC ACG TTC GGG TTT GAA GAA GAA GA	Linker sequence R -> F
hSqstm1 FEEED R	TC TTC TTC TTC AAA CCC GAA CGT GTC CT	Linker sequence R -> F
hSqstm1 WEEED F	AG GAC ACG TTC GGG TGG GAA GAA GAA GA	Linker sequence R -> W
hSqstm1 WEEED R	TC TTC TTC TTC CCA CCC GAA CGT GTC CT	Linker sequence R -> W
hSqstm1 N-term Flag F	gcc tcc cag gac acg ttc ggg gat tat aaa gat gat gat gat aaa gtg atc tgc gat ggc tgc aat	Linker sequence change to flag sequence
hSqstm1 N-term Flag R	attgcagccatcgcagatcactttatcatcatcatctttataatccccgaacgtgtcctgggaggc	Linker sequence change to flag sequence
hSqstm1 LEEED F	AG GAC ACG TTC GGG TTG GAA GAA GAA GA	Linker sequence R -> L
hSqstm1 LEEED R	TC TTC TTC TTC CAA CCC GAA CGT GTC CT	Linker sequence R -> L
hSqstm1 IEEED F	AG GAC ACG TTC GGG ATT GAA GAA GAA GA	Linker sequence R -> I
hSqstm1 IEEED R	TC TTC TTC TTC AAT CCC GAA CGT GTC CT	Linker sequence R -> I
hSqstm1 YEEED F	AG GAC ACG TTC GGG TAT GAA GAA GAA GA	Linker sequence R -> Y

hSqstm1 YEEED R	TC TTC TTC TTC ATA CCC GAA CGT GTC CT	Linker sequence R -> Y
hSqstm1 HEEED F	AG GAC ACG TTC GGG CAT GAA GAA GAA GA	Linker sequence R -> H
hSqstm1 HEEED R	TC TTC TTC TTC ATG CCC GAA CGT GTC CT	Linker sequence R -> H
hSQSTM1 I126A F	TAT TTT CAG GGA TCT gct TGC GAT GGC TGC AAT	Ile 126 to Ala 126 mutation
hSQSTM1 I126A R	ATT GCA GCC ATC GCA agc AGA TCC CTG AAA ATA	Ile 126 to Ala 126 mutation
hSqstm1 D129R F	GGA TCT GCT TGC cgt GGC TGC AAT GGG	Asp 129 to Arg 129 mutation
hSqstm1 D129R R	CCC ATT GCA GCC acg GCA AGC AGA TCC	Asp 129 to Arg 129 mutation
hSqstm1 R139D F	GTG GTA GGA ACC gac TAC AAG TGC AGC	Arg 139 to Asp 139 mutation
hSqstm1 R139D R	GCT GCA CTT GTA gtc GGT TCC TAC CAC	Arg 139 to Asp 139 mutation
hSqstm1 D147R F	AGC GTC TGC CCA cgt TAC GAC TTG TGT	Asp 147 to Arg 147 mutation
hSqstm1 D147R R	ACA CAA GTC GTA acg TGG GCA GAC GCT	Asp 147 to Arg 147 mutation
hSqstm1 D149R F	TGC CCA GAC TAC cgt TTG TGT AGC GTC	Asp 149 to Arg 149 mutation
hSqstm1 D149R R	GACGCTACACAAacgGTAGTCTGGGCA	Asp 149 to Arg 149 mutation
hSqstm1 KEEED F	AG GAC ACG TTC GGG AAA GAA GAA GAA GA	Linker sequence R -> K
hSqstm1 KEEED R	TC TTC TTC TTC TTT CCC GAA CGT GTC CT	Linker sequence R -> K
hSqstm1 D129N F	AAT GTG ATC TGC AAC GGC TGC AAT GGG	Asp 129 to Asn 129 mutation
hSqstm1 D129N R	CCC ATT GCA GCC GTT GCA GAT CAC ATT	Asp 129 to Asn 129 mutation
hSqstm1 PEEED F	AG GAC ACG TTC GGG CCG GAA GAA GAA GA	Linker sequence R -> P
hSqstm1 PEEED R	TC TTC TTC TTC CGG CCC GAA CGT GTC CT	Linker sequence R -> P
hSqstm1 GEEED F	AG GAC ACG TTC GGG GGC GAA GAA GAA GA	Linker sequence R -> G
hSqstm1 GEEED R	TC TTC TTC TTC GCC CCC GAA CGT GTC CT	Linker sequence R -> G
Ub BamHI F	tca gga tcc atg cag atc ttc gtg aag act ctg act	UB-R-BiP gene cloning
UB_R-Bip B R	ctt gtc ctc ctc ctc gcg ccc acc tct gag acg gag tac	UB-R-BiP gene cloning
UB_B-Bip C F	ctc cgt ctc aga ggt ggg cgc gag gag gag gac aag	UB-R-BiP gene cloning
R-Bip HindIII R (ns)	aat aag ctt caa ctc atc ttt ttc tgc tgt atc ctc ttc	UB-R-BiP gene cloning
LC3B_REEED_hSqstm1 S3 B_R	cac ggt gag cga atc ttc ttc ttc acg ccc gaa cgt ctc ctg gga ggc ata gac	LC3B_REEED_hSqstm1 generating
LC3B_REEED_hSqstm1 S3 C_F	gag acg ttc ggg cgt gaa gaa gaa gat tcg ctc acc gtg aag gcc tac ctt	LC3B_REEED_hSqstm1 generating

Sqstm1 N132L F	tgc gat ggc tgc ctt ggg cct gtg gta	Asn 132 to Leu 132 mutation
Sqstm1 N132L R	taccacaggcccaaggcagccatcgca	Asn 132 to Leu 132 mutation
Sqstm1 XhoI F	acg ctc gag atg gcg tcg ctc acc gtg aag gcc	Full-length gene cloning
Sqstm1 HindIII R	agc aag ctt caa cgg cgg ggg atg ctt tga ata	Full-length gene cloning
Sqstm1 SalI F	acg gtc acg atg gcg tcg ctc acc gtg aag gcc	Full-length gene cloning
Sqstm1 AgeI R	ctc acc ggt caa cgg cgg ggg atg ctt tga ata	Full-length gene cloning
Ub-R19 BiP HindIII F	acc aag ctt atg cag atc ttc gtg aag act cgt	UB-R19-BiP gene cloning
Ub-R19 BiP PstI R	agt ctg cag caa ctc atc ttt ttc cgt atc ctc	UB-R19-BiP gene cloning
Ub-R19 BiP PstI+1 F	tcc ctg cag a atg cag atc ttc gtg aag act cgt	UB-R19-BiP gene cloning
Ub-R19 BiP BamHI+2 R	agt gga tcc at caa ctc atc ttt ttc cgt atc ctc	UB-R19-BiP gene cloning
Ub-R19 BiP SacII R	agt ccg cgg caa ctc atc ttt ttc cgt atc ctc	UB-R19-BiP gene cloning
hSqstm1 H66A F	ggc ttc cag gcg gcc tac cgc gat gag	His 66 to Ala 66 mutation
hSqstm1 H66A R	ctc atc gcg gta ggc cgc ctg gaa gcc	His 66 to Ala 66 mutation
hSqstm1 H109A F	tgc cgg cgg gac gca cgc cca ccg tgt	His 109 to Ala 109 mutation
hSqstm1 H109A R	aca cgg tgg gcg tgc gtc ccg ccg gca	His 109 to Ala 109 mutation
Sqstm1 C113A F	c cac cgc cca ccg gct gct cag gag gcg c	Cys 113 to Ala 113 mutation
Sqstm1 C113A R	g cgc ctc ctg agc agc cgg tgg gcg gtg g	Cys 113 to Ala 113 mutation
hSqstm1 N 103 F	att aaa gag aaa aaa tag tgc cgg cgg gac	C-terminal deletion N103 generating
hSqstm1 N 103 R	gtc ccg ccg gca cta ttt ttt ctc ttt aat	C-terminal deletion N103 generating
hSqstm1 N121 F	ccc cgc aac atg tag cac ccc aat gtg	C-terminal deletion N121 generating
hSqstm1 N121 R	cac att ggg gtg cta cat gtt gcg ggg	C-terminal deletion N121 generating
hSqstm1 R21A F	gcg cgc gag att gcc cgc ttc agc ttc	Arg 21 to Ala 21 mutation
hSqstm1 R21A R	gaa gct gaa gcg ggc aat ctc gcg cgc	Arg 21 to Ala 21 mutation
hSqstm1 R22A F	cgc gag att cgc gcc ttc agc ttc tgc	Arg 22 to Ala 22 mutation
hSqstm1 R22A R	gca gaa gct gaa ggc gcg aat ctc gcg	Arg 21 to Ala 21 mutation
hSqstm1 E81A F	ttt tcc agt gac gcg gaa ttg aca atg	Glu 81 to Ala 81 mutation
hSqstm1 E81A R	cat tgt caa ttc cgc gtc act gga aaa	Glu 81 to Ala 81 mutation

hSqstm1 E82A F	tcc agt gac gag gca ttg aca atg gcc	Glu 82 to Ala 82 mutation
hSqstm1 E82A R	ggc cat tgt caa tgc ctc gtc act gga	Glu 82 to Ala 82 mutation
hSqstm1 N172 F	ctc gca ttc ccc tag ccc ttc ggg cac	C-terminal deletion N172 generating
hSqstm1 N172 R	gtg ccc gaa ggg cta ggg gaa tgc gag	C-terminal deletion N172 generating
